# Madelung disease with postoperative priapism and multiple venous thromboses: case report and literature review

**DOI:** 10.3389/fcvm.2024.1449556

**Published:** 2024-08-27

**Authors:** Linfen Guo, Wei Li, Xuewen Xu, Haitao Xiao

**Affiliations:** Department of Plastic and Burns Surgery, West China Hospital, Sichuan University, Chengdu, Sichuan, China

**Keywords:** Madelung disease, benign symmetric lipomatosis, liposuction, priapism, thrombosis

## Abstract

Madelung disease is an uncommon metabolic disorder of uncertain pathogenesis, distinguished by the symmetric accumulation of nonencapsulated adipose tissue within the subcutaneous layer of the neck, abdomen, thighs, and other anatomical regions. This condition has been tightly connected with comorbidities including diabetes, dyslipidemia, hyperuricemia, hypothyroidism, and adrenal dysfunction, as well as sensory, motor, and autonomic polyneuropathy. The prevalence of Madelung disease is conspicuously higher in Mediterranean and Eastern European, with a distinct scarcity within the Asian population. Surgical interventions involving lipectomy and liposuction represent the foremost and most efficacious treatment approach. Herein, we present a case encompassing type II Madelung disease featuring bilateral thighs adipose tissue accumulation. The patient exhibited unexplained priapism alongside multiple venous thrombosis during four surgical interventions. The infrequent manifestation of postoperative hypercoagulability in patients of Madelung disease merits broad attention, owing to the potentiality for extensive venous thrombosis and consequential severe outcomes such as pulmonary embolism or cerebral infarction arising from thrombus dislodgment. Building upon this clinical scenario, we systematically documented the clinical manifestations and disease progression in this patient, meticulously analyzed the causes of complications, and proposed targeted preventive measures. Additionally, we conducted a comprehensive review of the relevant literature to summarize the clinical and epidemiological features of Madelung disease and to elucidate its mechanisms. This study will provide a valuable reference for future clinical treatments and mitigate perioperative complications of Madelung disease.

## 1 Introduction

Madelung disease (MD), also recognized as benign symmetric lipomatosis (BSL), multiple symmetric lipomatosis (MSL), and Launois-Bensaude syndrome, is a sporadic and uncommon metabolic disorder (1). It was initially described by Brodie in 1846 and systematically defined by Madelung in 1888 (2). The hallmark of MD is the symmetric accumulation of nonencapsulated adipose tissue within the subcutaneous layer of anatomical regions such as the head, neck, shoulders, and other sites, resulting in distinctive physical features termed “hamster cheeks”, “horse collar” or “buffalo hump” (3, 4). The widely accepted classification proposed by Enzi delineates two anatomically based types of MD. Type I is the most common form, and predominantly involves symmetric deposition in the neck, shoulders, supraclavicular triangle, and proximal upper limbs, whereas type Ⅱ is characterized by deposits in the abdomen and thighs, occasionally resembling normal obesity (5). Notably, gender influences the occurrence of MD, with men often manifesting type I and women more exhibiting type II. 2161. In the 1990s, a four-fold classification introduced by Donhauser included neck distribution, pseudo-athletic appearance, gynoid presentation, and abdominal type (6). In addition, rare occurrences of adipose tissue deposits in unusual sites like breasts (7), tongue (8), and scrotum (9) have been documented. These clinical manifestations generally progress slowly, remain painless, and rarely exhibit malignancy (10). However, progressive neck adipose tissue accumulation may lead to serious complications such as neck stiffness, dysphagia, dysphonia, breathing difficulties, headache, and even sleep apnea syndrome (11). While MD can manifest across various ethnic groups, a preponderance of reported cases is notably observed within Mediterranean and Eastern European populations, particularly prevalent in regions such as Portugal and Italy (12). Relatively speaking, this condition demonstrates rarity within the Asian population (13). The exact prevalence and incidence of MD remain elusive, but estimates are available for certain countries. For example, the incidence of Italy males is approximately 1 in 25,000. It affects males more often with a male-to-female ratio of approximately 15:1–30:1, and 30–60 years appear to be the highest risk age of affection (14). The pathogenesis of MD remains incompletely understood. Current hypotheses suggest that mutations in mitochondrial DNA and a reduction in adrenergic-mediated lipolysis are primary contributing factors to the development of MD (15). The diagnosis of MD primarily relies on medical history, clinical features, and imaging modalities, particularly computed tomography (CT) or magnetic resonance imaging (MRI), to ascertain adipose tissue deposition (16). Differential diagnosis should encompass conditions such as morbid obesity, other types of lipomatosis, goiter, salivary gland disease, Cushing's disease, and neoplastic transformations (17). Given the absence of standardized guidelines, the management of MD varies under different conditions, encompassing health intervention, medication, lipolytic injection, and surgical procedures such as liposuction and lipectomy. Among these options, surgical interventions are considered as the foremost and most efficacious treatment modalities (18, 19). Herein, we present a case encompassing type II MD featuring bilateral thighs adipose tissue accumulation. Notably, the patient exhibited unexplained priapism alongside multiple venous thrombosis throughout the body during four surgical interventions. Building upon this clinical scenario, we systematically documented the clinical manifestations and disease progression in this patient, meticulously analyzed the causes of complications, and proposed targeted preventive measures. Additionally, we conducted a comprehensive review of the relevant literature to summarize the clinical and epidemiological features of MD and to elucidate its mechanisms. This study will provide a valuable reference for future clinical treatments and mitigate perioperative complications of MD.

## 2 Case presentation and data extraction

**Figure 1:**
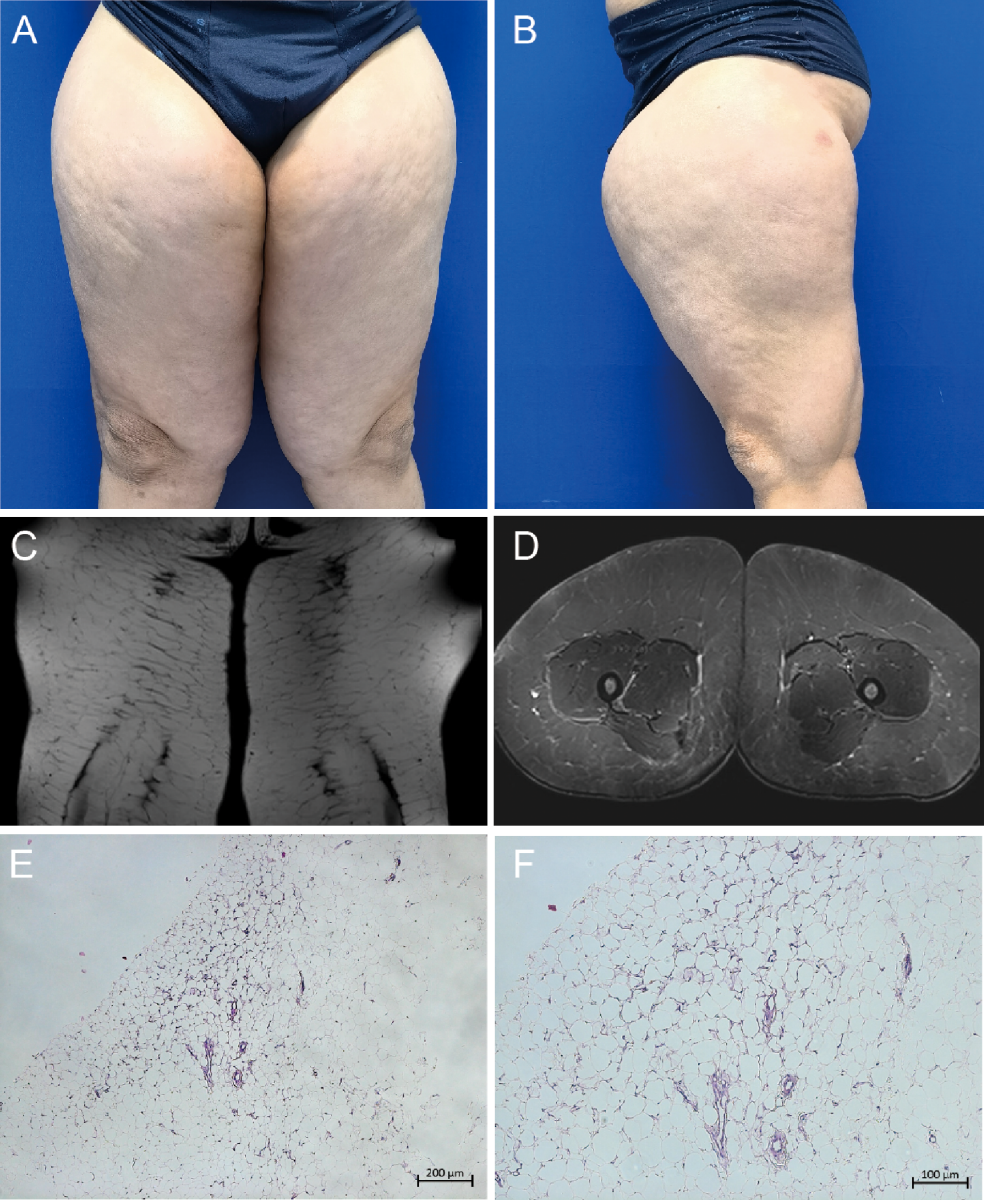
Phenotypic manifestations of Madelung disease. **(A, B)** Frontal and lateral view of bilateral thighs; **(C, D)** magnetic resonance imaging showing coronal and cross section of bilateral thighs; **(E, F)** histopathological assessment of the excised tissue revealing an adipogenic lesion.

A 49-year-old Chinese male presented with excessive thigh thickening, which first appeared 4 years ago and had significantly worsened over the past year (Figures 1A,B). The diffuse symmetric thickening spanned from the hip to the knee level, with a maximum diameter of 30 cm. The MRI suggested a significant thickening of the subcutaneous fat layer and adipose tissue filling within the muscle interstices, leading to a diagnosis of MD (Figures 1C,D). Administering 1.0 g of intravenous cefazolin sodium daily for three consecutive days perioperatively as preoperative prophylactic antibiotic treatment. Considering the substantial wound, the first liposuction surgery, as well as fascial and skin flap plasty, was limited to the patient's left thigh, popliteal fossa, knee, and hip. Approximately 4,000 ml of adipose tissue were successfully extracted. The risk of venous thromboembolism (VTE) was evaluated preoperatively and postoperatively using the Caprini assessment scale (20). The patient was assessed at intermediate risk postoperatively due to three risk factors, age 41–60 years, a body mass index greater than 25 kg/m^2^, and undergoing major surgery lasting over 45 min. Following the guidelines for VTE prophylaxis, the patient was administered 40 mg of enoxaparin sodium subcutaneously for two consecutive days. The histopathological assessment of the excised tissue revealed an adipogenic lesion without murine double minute 2 gene amplification, supporting the diagnosis of MD (Figures 1E,F).

**Figure 2:**
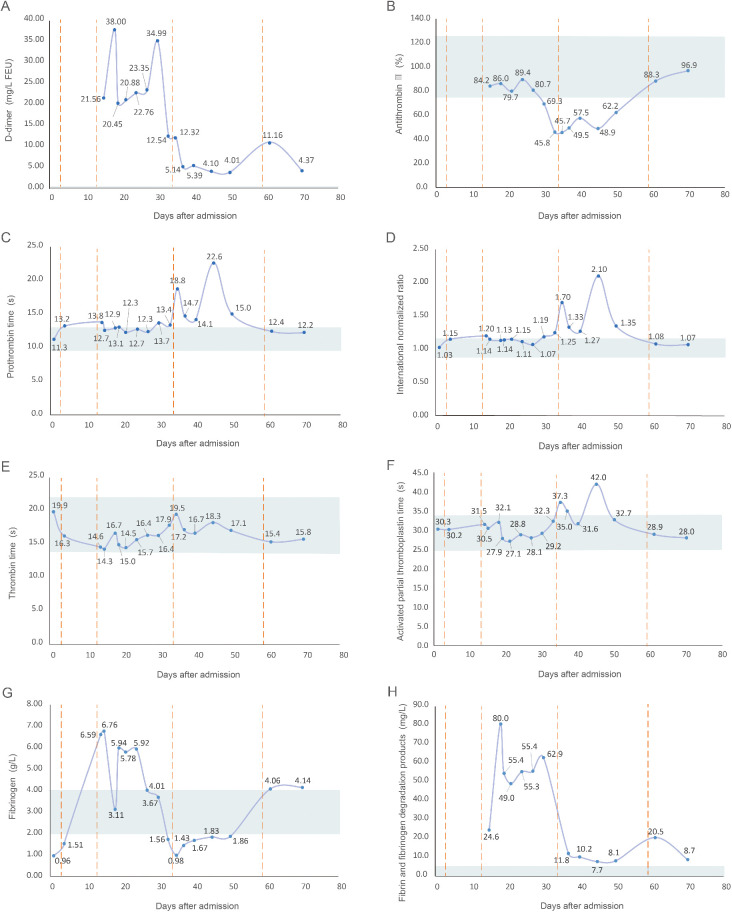
Line graph of alterations in coagulation indicators after the patient's admission. **(A)** D-dimer, with a reference range of <0.55 mg/L FEU; **(B)** antithrombin III, with a reference range of 75.0%–125.0%; **(C)** prothrombin time, with a reference range of 9.6–12.8 s; **(D)** international normalized ratio, with a reference range of 0.88–1.15; **(E)** thrombin time, with a reference range of 14.0–22.0 s; **(F)** activated partial thromboplastin time, with a reference range of 24.8–33.8 s; **(G)** fibrinogen, with a reference range of 2.00–4.00 g/L; **(H)** fibrin and fibrinogen degradation products, with a reference range of <5.0 mg/L. The shaded region denotes the reference range of coagulation indicators, the solid line illustrates value fluctuations in the coagulation indicators, and the dashed line represents the time of the patient's four surgeries after admission.

**Figure 3:**
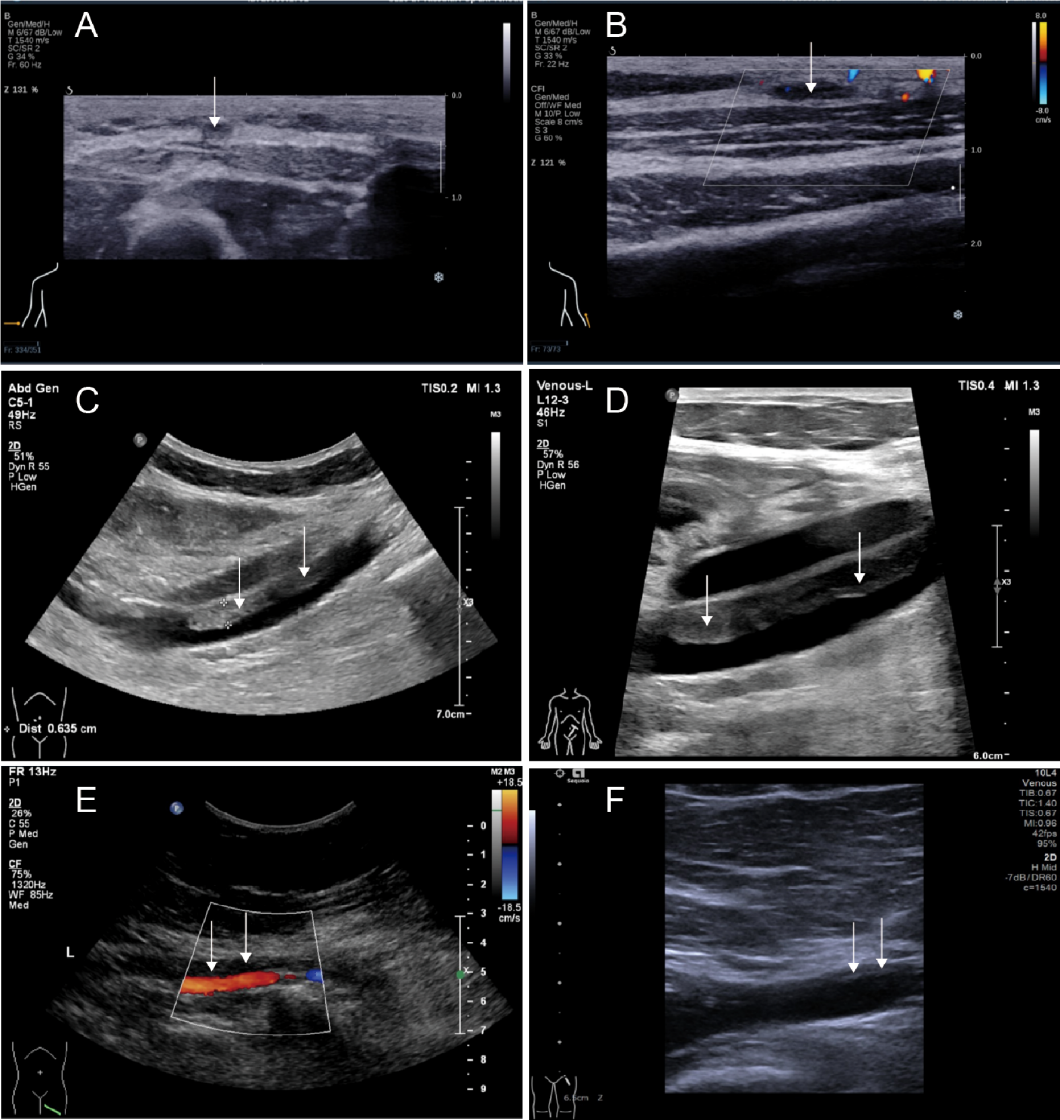
Ultrasonographic findings of multiple venous thromboses. **(A)** Right cephalic vein; **(B)** left cephalic vein; **(C)** right external iliac vein; **(D)** left external iliac vein; **(E)** left popliteal vein, **(F)** left common femoral vein.

After the liposuction surgery, the patient exhibited dark red coloration in the left thigh skin flap, along with epidermal detachment and scab formation. Concurrently, priapism and scrotal swelling were observed. Then left thigh debridement was conducted, and the patient experienced occasional exacerbation of priapism. Each instance of penile cavernous body suction and lavage provided slight relief. Penile and vascular ultrasound examinations showed no abnormalities, and the patient declined the penile head-cavernous body shunt operation. However, on the fourth day after the left thigh debridement, the patient's priapism symptoms dramatically worsened, accompanied by intolerable pain and significant hematuria. Laboratory blood tests revealed many indicator values surpassing the established reference compared with the results during hospitalization (Table 1). Alterations in indicators such as C-reactive protein and Interleukin-6, substantiated the progression of infection. Especially, the coagulation indicator tests revealed the D-dimer and fibrin and fibrinogen degradation products (FDP) levels surged to 38.00 mg/L FEU and 80.0 mg/L respectively (Figure 2). Lower abdominal computed tomography angiography (CTA) indicated right internal iliac vein embolism, while urological ultrasound demonstrated right hydronephrosis and ureteral dilatation. Subsequent bladder irrigation and anticoagulant treatment led to partial improvement in the patient's condition. The anticoagulant treatment strategy was continuously adjusted based on Caprini assessment results, blood coagulation indicators, and imaging findings. Initially, 60 mg of enoxaparin sodium was administered for four consecutive days, followed by 60 mg of nadroparin calcium for 16 consecutive days. After stabilization, 60 mg of edoxaban tosilate tablets were administered orally daily for 2 months. Additionally, wound area secretion culture suggested successive infections with Escherichia coli, Acinetobacter baumannii, and Klebsiella pneumoniae. Intravenous administration of ceftriaxone sodium, tigecycline, imipenem-cilastatin sodium, and colistin sulfate, along with the topical application of silver sulfadiazine, constituted the spectrum of antibiotics employed in practice. During anticoagulation therapy, the extremity venous ultrasound suggested the development of bilateral cephalic vein thrombosis (Figures 3A,B), along with left common femoral vein reflux and bilateral calf soft tissue swelling. Abdominal venous ultrasound revealed bilateral external iliac venous mural thrombus (Figures 3C,D). Subsequently, the patient underwent skin grafting for the traumatized area of the left thigh, with the donor areas being successively the right thigh and scalp. Follow-up extremity venous ultrasound revealed left popliteal vein thrombosis and left common femoral vein thrombosis (Figures 3E,F). When the patient was discharged from the hospital 10 days after the fourth surgery, most of the skin graft pieces exhibited good viability and the peritraumatic flap demonstrated an appropriate fit. Additionally, an improvement in priapism and swelling was observed. The patient was advised to continue oral anticoagulant therapy, with regular monitoring of coagulation function and venous ultrasound reviews.

**Table 1:** Results of blood tests during hospitalization and at the exacerbation of abnormal priapism.

Variable	At admission		At the exacerbation		Reference range	
	Value	Trend	Value	Trend	Value	Unit
Infection indicators						
C-reactive protein	—	—	118	↑	<5.00	mg/L
Interleukin-6	—	—	164	↑	0.00–7.00	pg/ml
Procalcitonin	—	—	1.56	↑	<0.046	ng/ml
Hemocyte analysis						
Erythrocyte count	4.43	→	3.17	↓	4.3–5.8	×10^12^/L
Hemoglobin	133	→	95	↓	130–175	g/L
Hematocrit	0.40	→	0.30	↓	0.40–0.50	L/L
Platelet count	166	→	262	→	100–300	×10^9^/L
Leukocyte count	5.43	→	24.56	↑	3.5–9.5	×10^9^/L
Neutrophilic segmented granulocyte,%	62.5	→	88.6	↑	40–75	%
lymphocyte,%	27.8	→	3.9	↓	20–50	%
Monocyte,%	7.4	→	7.2	→	3–10	%
Eosinophil,%	1.7	→	0.1	↓	0.4–8.0	%
Basophil,%	0.6	→	0.2	→	0.0–1.0	%
Neutrophilic segmented granulocyte	3.39	→	21.76	↑	1.8–6.3	×10^9^/L
Lymphocyte	1.51	→	0.96	↓	1.1–3.2	×10^9^L
Monocyte	0.40	→	1.77	↑	0.1–0.6	×10^9^/L
Eosinophil	0.09	→	0.02	→	0.02–0.52	×10^9^/L
Basophil	0.03	→	0.05	→	0.00–0.06	×10^9^/L
Biochemical indicators						
Total bilirubin	8.7	→	20.3	→	5.0–28.0	umol/L
Direct bilirubin	2.4	→	13.2	↑	<8.8	umol/L
Indirect bilirubin	6.3	→	7.1	→	<20	umol/L
Total bile acid	5.0	→	8.1	→	<15	umol/L
Alanine aminotransferase, ALT	14	→	141	↑	<50	IU/L
Aspartate aminotransferase, AST	14	→	86	↑	<40	IU/L
AST/ALT ratio	1.00		0.61			
Alkaline phosphatase	70	→	443	↑	51–160	IU/L
Glutamyl transpeptidase	20	→	443	↑	<60	IU/L
Total protein	65.3	→	56.4	↓	65.0–85.0	gL
Albumin, ALB	43.4	→	33.5	↓	40.0–55.0	g/L
Globulin, GLOB	21.9	→	22.9	→	20.0–40.0	g/L
ALB/GLOB ratio	1.98	→	1.46	→	1.24–2.40	
Glucose	4.71	→	9.10	↑	3.90–5.90	mmol/L
Urea	5.3	→	5.2	→	3.1–8.0	mmol/L
Creatinine	71	→	87.00	→	68–108	umol/L
Estimated glomerular filtration rate	104.73		89.70			ml/min/1.7
Cystatin-C	1.06	→	1.44	↑	0.51–1.09	mg/L
Uric acid	344	→	176	↓	240–290	umol/L
Triglyceride	0.76	→	0.93	→	0.29–1.83	mmol/L
Total cholesterol	4.70	→	3.63	→	2.80–5.70	mmol/L
High density lipoprotein cholesterol	1.52	→	0.80	↓	>0.90	mmol/L
Low density lipoprotein cholesterol	2.86	→	2.48	→	<4.0	mmol/L
Non-high density lipoprotein cholesterol	3.18		2.83			mmol/L
Creatine kinase	42	→	27	→	19–226	IU/L
Lactic dehydrogenase	131	→	288	↑	120–150	IU/L
Hydroxybutyrate dehydrogenase	101	→	211	↑	72–182	IU/L
Sodium	140.8	→	125.4	↓	137.0–147.0	mmol/L
Potassium	3.89	→	4.22	→	3.50–5.30	mmol/L
Chlorine	106.3	→	89.1	↓	99.0–110.0	mmol/L
Calcium	2.25	→	2.01	↓	2.11–2.52	mmol/L
Magnesium	0.89	→	0.72	↓	0.75–1.02	mmol/L
Inorganic phosphorus	1.03	→	0.96	→	0.85–1.51	mmol/L

The symbols employed for indicating blood test outcomes are as follows: “—” signifies the non-availability of the corresponding laboratory measurement; “→” signifies the test value falls within the predefined reference range; “↑” signifies the test value exceeds the predefined reference range; and “↓” signifies the test value is below the predefined reference range.

## 3 Discussion

The underlying mechanisms driving the pathogenesis of MD remain unclear. A prominent hypothesis posits that MD's initiation is underpinned by the tumor-like proliferation of brown adipose tissue, a phenomenon closely tied to the diminished lipolysis stemming from reductions in β-adrenergic receptor numbers or activity (15). Additionally, perturbations in the mitochondrial function of adipose tissues constitute an integral facet, encompassing compromised respiratory chain integrity and attenuated cytochrome C oxidase activity (21, 22). In MD patients associated with lipodystrophic syndrome, the MNF2 gene is mutated and exhibits an autosomal recessive form, with the causative variant p.Arg707Trp in the homozygous or composite heterozygous state (23). This gene encodes mitofusin-2, which is involved in mitochondrial fusion and endoplasmic reticulum-mitochondrial interactions (24). Defects in MNF2 result in uncoupling protein 1-negative unilocular adipocytes with enlarged and disorganized mitochondria, reduced mitochondrial DNA levels, increased expression of mitochondrial oxidative stress-related genes, and a marked decrease in leptin and lipocalin expression (25). Moreover, in a family presenting with myopathy and lipodystrophic syndrome, a phenotype resembling MD in non-dystrophic regions was linked to the mutation of the homozygous Lipase E gene, which encodes hormone-sensitive lipase, a key enzyme in triglyceride metabolism (26). Beyond these Mendelian forms, studies suggest that MD shows detectable mitochondrial DNA pathogenic variants, such as MT-TK, pos.8344A > G, and m.8357T > C (27, 28). These identified genes could represent potential therapeutic targets. While the definitive mechanism is elusive, alcoholism emerges as a discernible catalyst for MD advancement (29). Patients with a chronic history of alcohol consumption comprise 60%–90% of cases and frequently suffer from secondary liver dysfunction and cirrhosis (30, 31). In addition, MD has also been tightly connected with the presence of several metabolic disorders, encompassing diabetes, dyslipidemia, hyperuricemia, gynecomastia, hypothyroidism, and adrenal dysfunction (3, 30, 32). Besides, sensory, motor, and autonomic polyneuropathy are concurrently identified in approximately 85% of individuals afflicted with MD (33). In accordance with Enzi's classification system (5), the presented case corresponds to type II. The feature of local adipose tissue accumulation of bilateral thighs renders differentiation from alternative forms of obesity or neoplastic conditions more straightforward. This patient's long-term history of alcohol abuse, along with risk factors such as gender, age, and obesity, likely played a significant role in the progression of MD. Especially, the patient's familial background is noteworthy, given that both parents are close relatives and his sister exhibited comparable clinical manifestations. This familial aggregation potentially furnishes an avenue for understanding the pathogenesis of MD.

Therapeutic approaches for MD encompass nonsurgical and surgical modalities. Nonsurgical interventions offer a limited therapeutic impact. While alcohol cessation and weight reduction may decelerate adipose mass enlargement and restore metabolic equilibrium to a relative extent, their ability to halt disease progression and effectuate reversal remains uncertain (34). Furthermore, effective and definitive pharmacotherapy for MD has yet to be established. In select reports, intralipotherapy involving the injection of phosphatidylcholine or deoxycholate has been proposed as a noninvasive intervention. Likewise, although this method effectively restrains adipose mass growth, its capacity to reduce volume is less corroborated (35). Besides, while studies have advocated the use of β2-agonist salbutamol for lipolysis induction through adrenergic stimulation, the clinical efficacy of such conservative approaches remains contentious (7, 33, 36). Surgical management aims to address cosmetic deformities, alleviate compressive symptoms, and safeguard crucial vascular and neural structures. Empirical evidence indicates surgical intervention as the foremost and most efficacious treatment modality, including lipectomy, liposuction, or their combination (19). Lipectomy predominates among reported cases, facilitating thorough exposure and meticulous excision with decreased inadvertent damage to contiguous structures. However, complete lipectomy presents technical challenges due to the absence of a discernible capsule demarcating the lipomatous tissue (37). Liposuction's rising popularity stems from its low complication morbidity, simplicity, lesser invasiveness, and enhanced cosmetic outcomes (19). Nevertheless, liposuction is considered as an adjunct therapy in numerous instances, due to the formidable density and fibrous composition of the adipose mass (38). Moreover, achieving comprehensive removal, whether through lipectomy or liposuction, proves intricate given the nonencapsulated nature, multidirectional expansion, and potential to involve diverse structures, rendering recurrence nearly inevitable (39). In conclusion, both lipectomy and liposuction offer merits and limitations. Surgeons should judiciously select the optimal therapies based on disease localization and extent, patient expectations, and the surgeon's proficiency. In consideration of the extensive adipose tissue infiltration, and relatively diminished vascular and neural structures around the thighs, left thigh liposuction was undertaken, aiming to minimize procedural trauma and postoperative complications. Regrettably, postoperative priapism and thrombosis emerged. Therefore, deferral of right thigh liposuction was deemed prudent.

The patient's hospitalization spanned a total of 72 days, during which he exhibited abnormal priapism and discernible swelling of the scrotal region for more than 2 months. In addition, the patient had previously encountered urinary difficulties devoid of evident causation. A possible explanation was that the patient had related pathological conditions like abnormal neurological function or hormone secretion, and surgical stress, trauma, and urinary tract infection triggered intractable priapism. In addition to the priapism, the patient experienced postoperative multiple venous thromboses distributed throughout the body which indicated a heightened propensity for an *in vivo* hypercoagulable state at this juncture. This perilous state then gradually waned around the third operation in response to appropriate and continuously adjusted anticoagulant therapy. Therefore, the patient did not develop symptoms, such as dyspnea or chest pain, nor the corresponding imaging manifestations of pulmonary embolism. The mechanism underlying multiple venous thromboses across various anatomical regions can be delineated as follows. First, the preoperative coagulation status was not sufficiently evaluated, and the postoperative venous ultrasound of the abdomen and limbs was not promptly addressed. Second, the liposuction surgery was characterized by prolonged duration, wide-range lesion involvement, and substantial surgical trauma. In response to the postoperative epidermal detachment, the patient underwent a second debridement surgery. Thus, anticoagulation was carefully managed during both surgeries to prevent postoperative bleeding. Third, the patient's adipose accumulation was primarily concentrated in both thighs, exerting prolonged compression on the blood vessels. It may have impacted the morphology and function of the lower extremity veins, with the defect becoming fully apparent post-surgery. Fourth, the patient's prolonged bedridden state after the surgery decelerated venous flow rates. Fifth, despite prompt support therapy of fluid replacement, the imbalance between exudation and relatively insufficient intake could potentially lead to blood concentration. Last, infection contributed to thrombosis by inducing the increased production of procoagulant compounds such as thrombin. Similarly, trauma, including major surgery, stimulated the release of proinflammatory cytokines and disrupted the regulation of tissue factor and thrombin, leading to a prothrombotic state (40). Evidence suggested that patients hospitalized in septic states faced a higher risk of VET (41). Consequently, the surgical trauma, along with the postoperative infectious state and septic predisposition, promoted the development of VET.

According to the International Society of Aesthetic Plastic Surgery Global Survey Result, liposuction was the most common surgical procedure in 2023 as in 2022, with more than 2.2 million (https://www.isaps.org/discover/about-isaps/global-statistics/reports-and-press-releases/global-survey-2023-full-report-and-press-releases/, accessed on July 29, 2024). A systematic review investigating the safety of large-volume liposuction revealed that the incidence of pulmonary embolism ranks second only to blood loss requiring transfusion among major surgical complications (42). Moreover, pulmonary thromboembolism is the most frequent major complication that can lead to death in patients undergoing liposuction (43). Therefore, it is imperative for plastic surgeons to thoroughly assess each MD patient's risk factors for deep vein thrombosis and formulate a comprehensive preventive strategy before performing liposuction. Instances of postoperative hypercoagulability leading to thromboses in patients with MD are relatively infrequent. A recent study documented a MD patient displayed thromboses in the local intermuscular veins of the bilateral calf and the left posterior tibial vein, along with multiple thromboembolisms in pulmonary arteries following the second cervical surgery (44). In another case, a patient manifested painful pressure-induced swelling and stasis dermatitis in the left calf initially presumed to signify deep vein thrombosis. However, substantial fatty infiltrative anomalies were discerned in his left calf and bilateral thighs through CTA. Consequently, this patient was finally diagnosed with MD, along with adipose tissue accumulation leading to deep venous compression (45). Hence, vigilance is essential to discern thrombosis or embolism symptom following MD surgery, with CT or MRI serving as pivotal tools for differential diagnosis. Preventive measures in accordance with anticoagulation standards must be diligently adhered to. First, the preoperative evaluation should comprehensively assess coagulation status and thrombotic risk, wherein coagulation indicators like D-dimer and imaging modalities like lower extremity venous ultrasonography should be promptly addressed. Second, vigilant monitoring of vital signs, localized status of lower limbs, pulmonary indications, as well as fluid balance variations is imperative. Third, patients should be educated to minimize bedridden periods, engage in timely mobilization, and consider the use of elastic stockings. Last, establishing a multidisciplinary collaborative approach involving hematology, interventional medicine, and other pertinent departments, and adjusting anticoagulation strategies tailored to the individual patient's evolving condition is suggested.

## 4 Conclusion

In this study, we present a case of type II MD featuring bilateral thighs adipose tissue accumulation. The patient exhibited unexplained priapism alongside multiple venous thromboses after the liposuction surgery. The infrequent manifestation of postoperative hypercoagulability in MD patients merits broad attention, owing to the potentiality for extensive venous thromboses and subsequent pulmonary embolism or cerebral infarction. Comprehensive assessment of coagulation risk assumes paramount significance in enhancing the prognosis of MD patients, with coagulation indicator surveillance and recurrent venous ultrasonography serving as pivotal tools for risk appraisal. Vigilant postoperative monitoring of vital signs, localized status of lower limbs, pulmonary indications, and fluid balance alterations, as well as proactive guidance to encourage early mobilization and the utilization of elastic stockings, holds substantial preventive value. Finally, the direct correlation between MD and the observed priapism or hypercoagulability necessitates further observation. Future studies describing relevant clinical cases for expanded comprehension are necessary.

## Data Availability

The original contributions presented in the study are included in the article/Supplementary Material, further inquiries can be directed to the corresponding author.
